# A Case Report of Metastatic Melanoma in the Transverse Colon

**DOI:** 10.7759/cureus.80659

**Published:** 2025-03-16

**Authors:** Sophia Bee Ting Tan, Michael Lamparelli

**Affiliations:** 1 General Surgery, Rockhampton Hospital, Rockhampton, AUS

**Keywords:** gastrointestinal malignant melanoma, gastrointestinal perforation, immunotherapy-related adverse events, metastatic melanoma, non-cutaneous malignant melanoma

## Abstract

Colonic metastasis from melanoma is extremely rare and is often asymptomatic, complicating detection. Diagnosis requires imaging and endoscopic evaluation. Treatment typically involves surgical resection with systemic therapies like immunotherapy and targeted therapy. However, immunotherapy has increased immune-related adverse events, including gastrointestinal perforation, a rare but serious complication. This report details a case of a Caucasian male patient in his 60s with a history of excised scalp melanoma eight years prior, who developed metastatic melanoma in the transverse colon. After initiating combination immunotherapy with ipilimumab/nivolumab, he suffered a bowel perforation, necessitating palliative care. The case underscores the need for vigilant monitoring for asymptomatic gastrointestinal tract metastases in melanoma patients and careful risk assessment when considering immunotherapy. It emphasizes the challenge of balancing aggressive treatment with managing potentially severe adverse events.

## Introduction

Metastatic melanoma is the most common malignancy to spread to the gastrointestinal tract (GIT), followed by breast and lung cancer [1]. While post-mortem studies estimate gastrointestinal (GI) involvement in up to 60% of patients with advanced melanoma, symptomatic disease occurs in only 1-5% of cases, with antemortem diagnosis remaining rare [1-3]. Colonic metastases are particularly uncommon, with reported incidences ranging from 0.18% to 2.1% [4]. Although melanoma can metastasize to any part of the GIT, the small bowel remains the most common site of GI metastasis, followed by the stomach, rectum, and colon [1]. Given its typically asymptomatic or nonspecific presentation, colonic metastases are often diagnosed late, delaying management and limiting treatment options. The interval between primary melanoma diagnosis and GI metastasis is highly variable, sometimes occurring years after initial treatment [5].

The differential diagnosis of colonic melanoma includes primary colorectal adenocarcinoma, lymphoma, neuroendocrine tumors, and GI stromal tumors, all of which require histopathological and immunohistochemical differentiation. Diagnosis relies on imaging modalities such as contrast-enhanced computed tomography (CT) and positron emission tomography (PET) scans, which help identify metastatic lesions, as well as colonoscopy with biopsy for definitive histological confirmation [2]. Melanoma markers such as SOX10, HMB-45, Melan-A, and S100 help distinguish metastatic melanoma from primary colonic malignancies [6].

Management of metastatic melanoma with GI involvement requires a combination of systemic therapy, surgical intervention for complications, and supportive care [1,7]. Immune checkpoint inhibitors (ICI) such as nivolumab and pembrolizumab have transformed melanoma treatment, often used alone or in combination with ipilimumab in metastatic disease. Targeted therapies, including BRAF and MEK inhibitors, are considered in BRAF-mutant melanoma [1]. While surgery is typically reserved for palliation in symptomatic patients with bleeding, obstruction, or perforation, select cases with isolated metastases may benefit from resection [8].

Despite therapeutic advancements, complications remain a significant concern. Colonic melanoma metastases can cause bowel obstruction, perforation, and GI bleeding [3,9]. The increasing use of ICIs has also led to a rise in immune-related adverse events, including severe colitis and spontaneous bowel perforation, as seen in this case. The underlying mechanisms of immunotherapy-induced perforation remain unclear, necessitating careful patient selection, close monitoring, and prompt intervention. Given the challenges in early detection and the risk of treatment-related complications, a high index of suspicion and an individualized management approach are crucial in optimizing patient outcomes.

## Case presentation

A Caucasian male patient in his 60s was referred by his general practitioner to our general surgical clinic with symptoms of abdominal pain, night sweats, and weight loss. His medical history indicated that he underwent excision of a melanoma on his scalp eight years ago. The initial melanoma was staged as pT1b based on histopathological findings, which revealed no ulceration, lymph-vascular invasion, perineural invasion, or satellite nodules. Regression was present in the papillary dermis, and tumor-infiltrating lymphocytes were classified as non-brisk. The tumor demonstrated a horizontal growth phase with a mitotic index of 1 per mm². It was identified as malignant melanoma, primarily in situ with focal superficial invasion, with a Breslow depth of 0.55 mm. A postoperative PET scan at the time showed no evidence of metastatic disease.

His past medical history also included a history of renal calculi, diverticular disease, and bilateral carpal tunnel syndrome.

Upon examination, the patient exhibited extensive sun-damaged skin; however, his general appearance was good. The physical examination identified a significant palpable mass in his right upper quadrant, without any indications of obstruction.

Investigations

**Figure 1:**
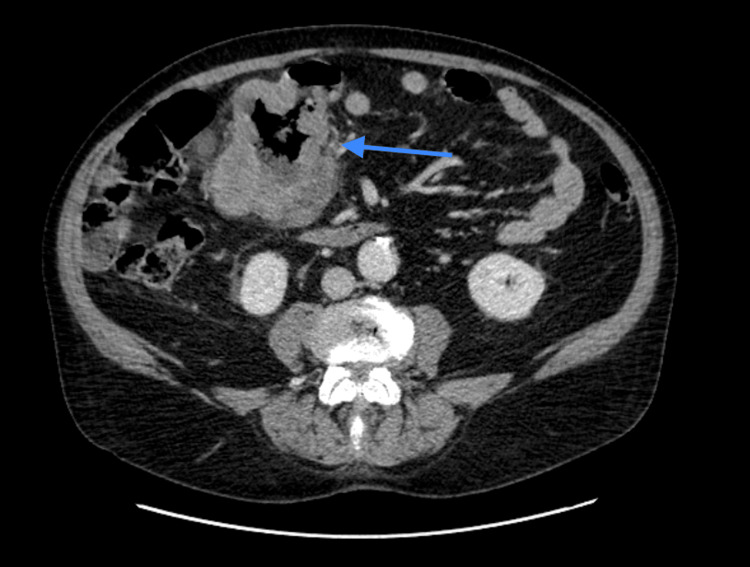
CT abdomen and pelvis with intravenous contrast (axial view) demonstrating significant irregular thickening within a 10 cm segment of the proximal transverse colon (arrow).

**Figure 2:**
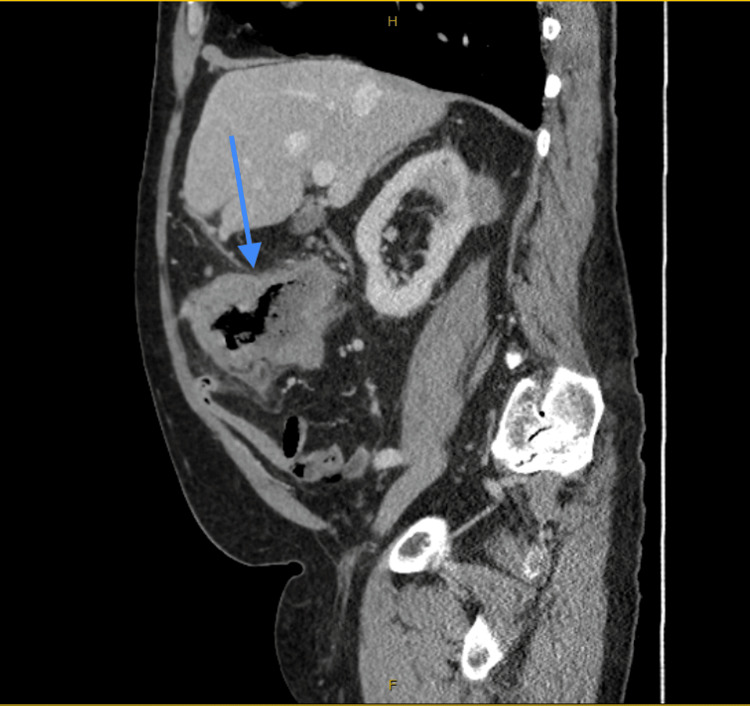
CT abdomen and pelvis with intravenous contrast (sagittal view) demonstrating significant irregular thickening within a 10 cm segment of the proximal transverse colon (arrow).

**Figure 3:**
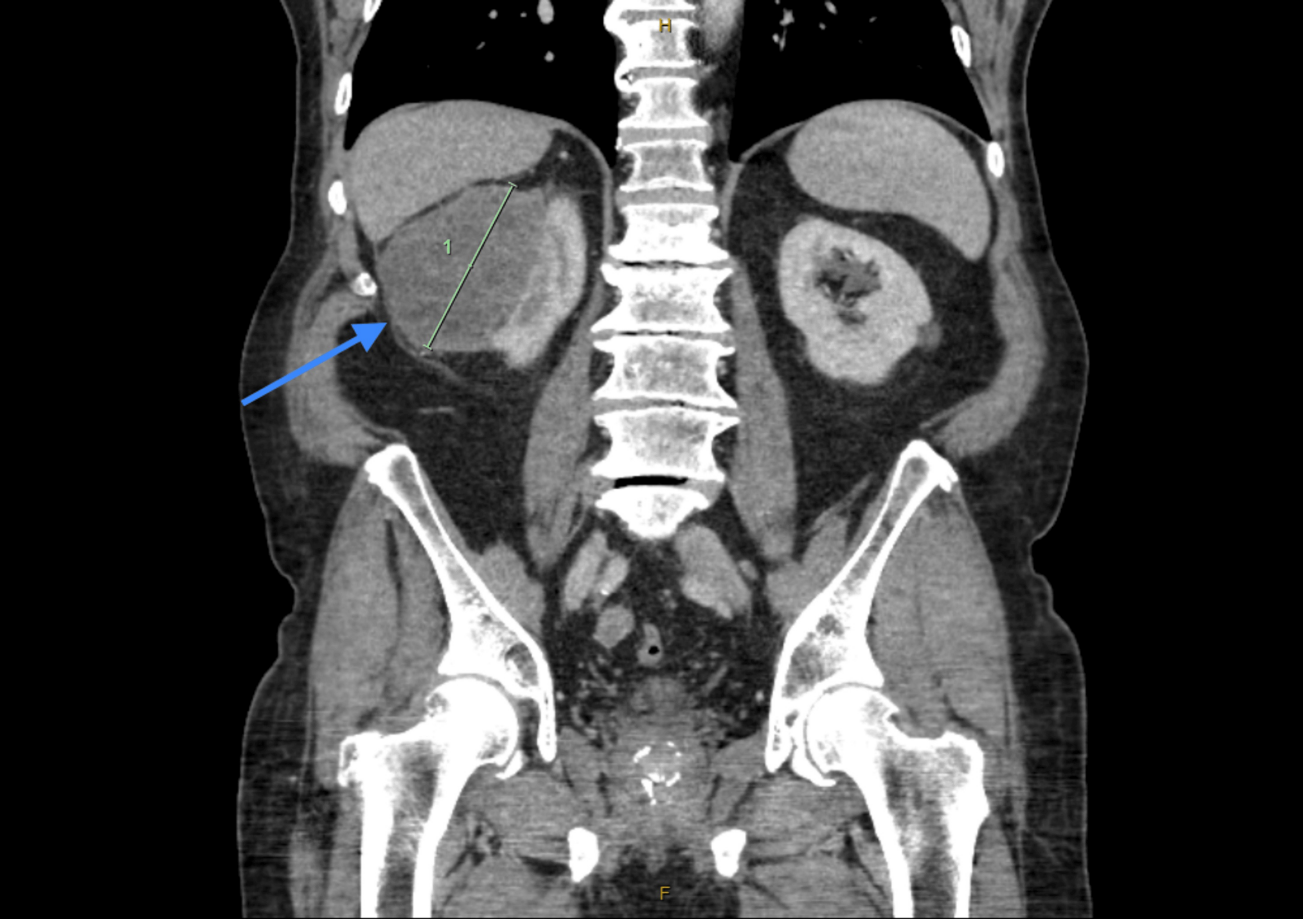
CT abdomen and pelvis with intravenous contrast demonstrating 96 mm mass in the right kidney (arrow).

**Figure 4:**
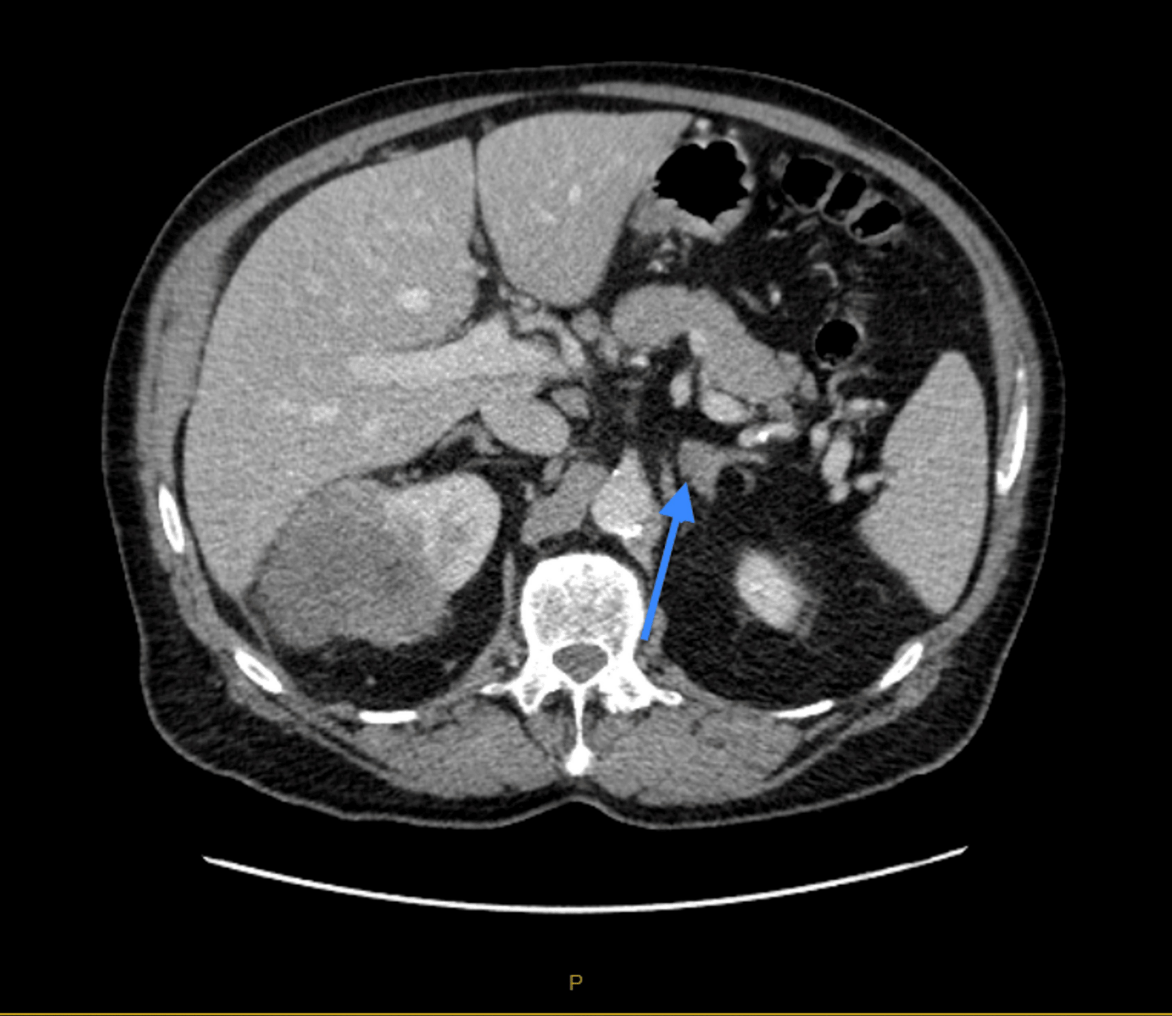
CT abdomen and pelvis with intravenous contrast demonstrating 15 mm nodule on the left adrenal gland (arrow).

CT of the abdomen and pelvis with contrast demonstrated significant irregular thickening within a 10 cm segment of the proximal transverse colon (Figure 1, Figure 2), accompanied by a 96 mm mass in the right kidney (Figure 3). A 15 mm nodule on the left adrenal gland raised suspicions for metastasis (Figure 4), given the clinical context. Biochemical assays, including liver function tests, lactate dehydrogenase, and tumor markers such as carcinoembryonic antigen (CEA), cancer antigen (Ca) 19.9, and Ca 125, were within normal limits. A subsequent CT for staging revealed no evidence of pulmonary masses or intrathoracic lymphadenopathy.

**Figure 5:**
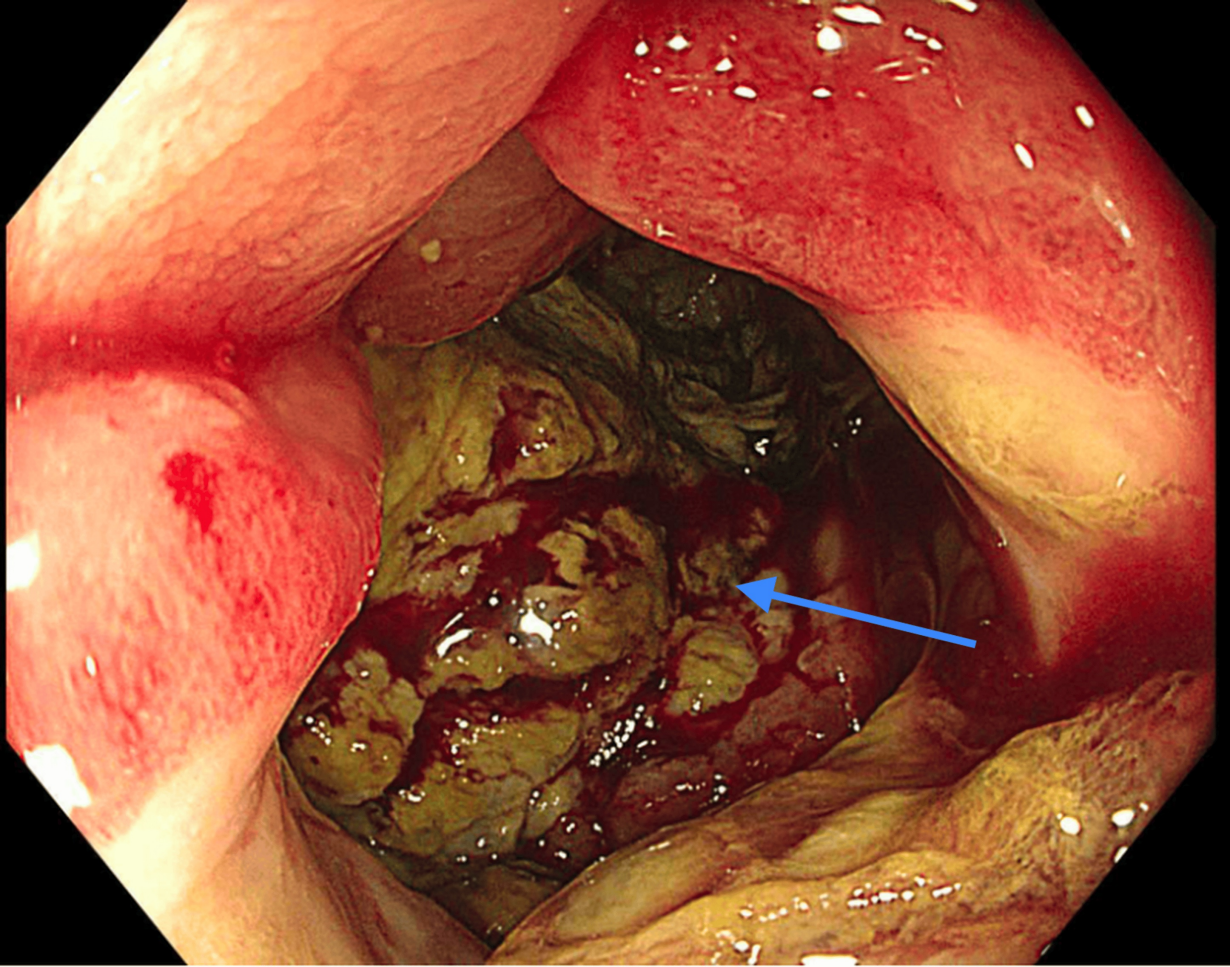
Colonoscopy showing circumferential, infiltrative, partially obstructing large mass in the transverse colon (arrow). The unusual tissues were very friable.

**Figure 6:**
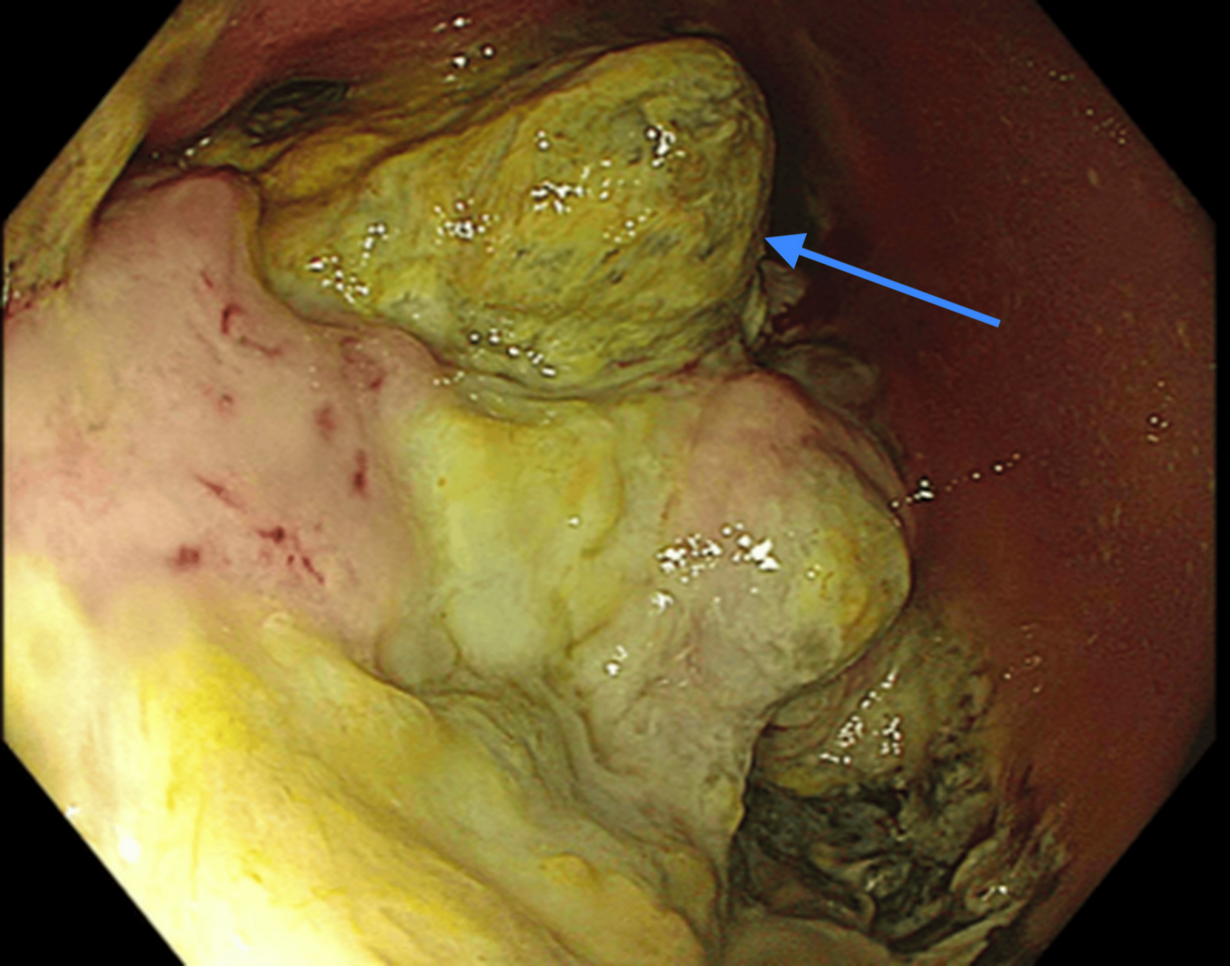
Colonoscopy showing circumferential, infiltrative, partially obstructing large mass in the transverse colon (arrow). The unusual tissues were very friable.

Colonoscopic evaluation disclosed a 10 cm infiltrative, circumferential and partially obstructing mass in the transverse colon, which was bleeding (Figure 5, Figure 6).

**Figure 7:**
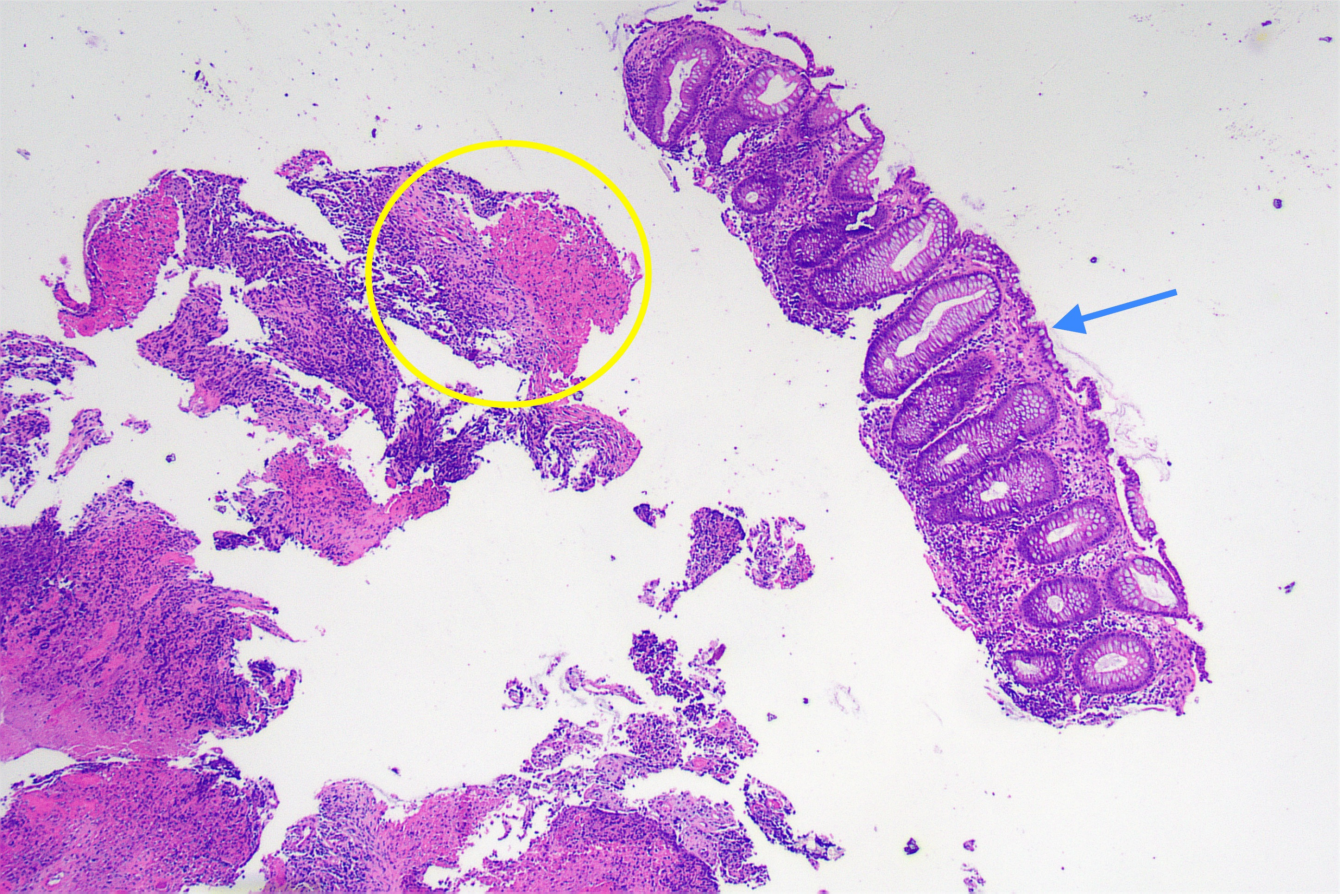
H&E micrograph x40. A low-power view shows a fragment of intact non-dysplastic colonic mucosa (arrow) and fragments of ulcerated tissue with variable cellularity (circle).

**Figure 8:**
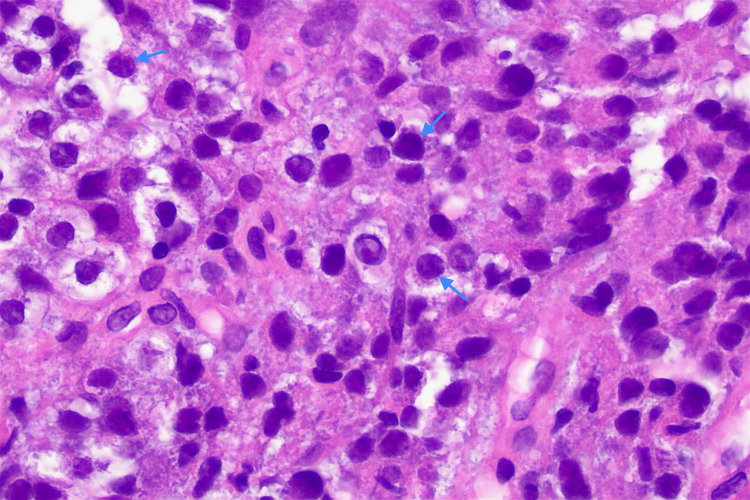
H&E micrograph x400. At high power, the cells have coarse chromatin, irregular outlines and occasional prominent nucleoli (arrow).

On histological examination, the biopsy specimen was revealed to consist of multiple fragments of ulcer base and unoriented fibrous tissue, together with a single intact fragment of colonic mucosa. The colonic mucosa showed no evidence of dysplasia. Ulcerated fragments showed variable cellularity imparted by a patchy infiltrate of atypical epithelioid cells with hyperchromatic nuclei and moderate amphophilic to clear cytoplasm. The atypical cells were dispersed in patternless sheets with no evidence of gland formation (Figure 7, Figure 8). On immunohistochemistry, the atypical cells showed diffuse, strong positivity for melanoma markers including SOX10, HMB-45, Melan-A, and S100. There was patchy, weak staining of some atypical cells with AE1/3. CK20 and CDX2 were negative. Melanoma markers highlighted further atypical cells in the deep edge of the mucosal fragment. The findings are consistent with malignant melanoma.

A comprehensive PET scan highlighted a highly fluorodeoxyglucose (FDG)-avid, enlarged right kidney, with additional uptake in the right upper quadrant bowel, peritoneum, and right pelvic peritoneum, indicative of metastasis. The FDG-negative left adrenal nodule is suggestive of a benign adenoma. An extensive panel of urine and blood tests, ordered to assess this adrenal incidentaloma, returned normal results, supporting the diagnosis of a benign adenoma. To rule out the possibility of a simultaneous primary malignancy, such as renal cell carcinoma, a biopsy of the right kidney is being scheduled.

Treatment

While awaiting additional diagnostic procedures, the patient was referred to a medical oncologist. Despite experiencing weight loss and systemic symptoms, the patient remained ambulatory and independent in daily activities, with an Eastern Cooperative Oncology Group (ECOG) status of 1. The decision to commence ICI therapy before surgery was based on the extent of metastatic disease. Given the widespread involvement, curative surgical resection was not a feasible option, and systemic therapy was prioritized to control disease progression. The colonic metastasis, while infiltrative, was not causing acute obstruction at presentation, allowing time for immunotherapy initiation. Surgical intervention was considered only if obstruction or complications arose. The proposed treatment strategy involved commencing with a shortened regimen of two cycles of induction immunotherapy using a combination of ipilimumab and nivolumab, instead of the standard four cycles, to minimize the potential for adverse effects. Subsequently, the patient would continue with maintenance therapy using nivolumab.

Outcome and follow-up

**Figure 9:**
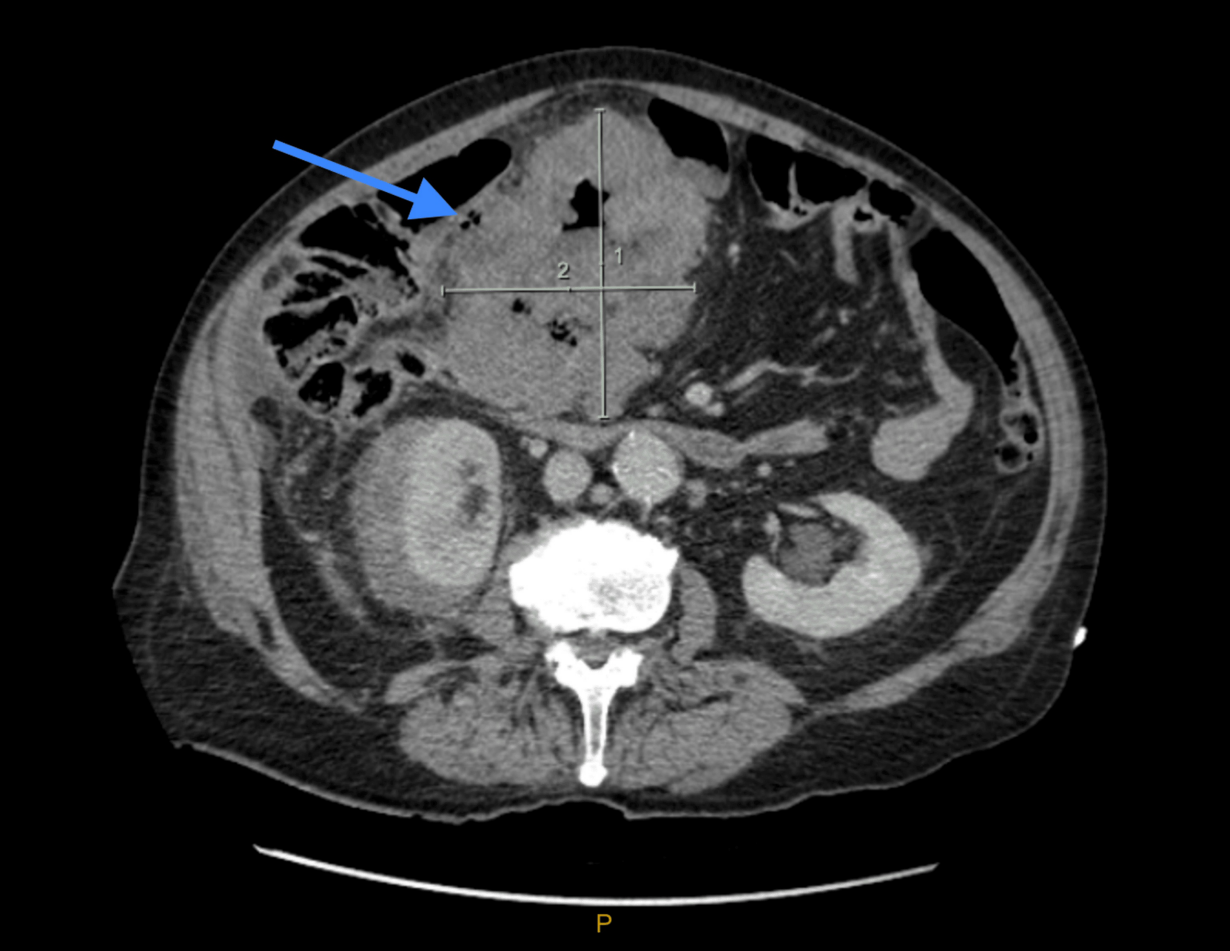
CT abdomen and pelvis with intravenous contrast demonstrating bowel perforation with pneumoperitoneum (arrow).

**Figure 10:**
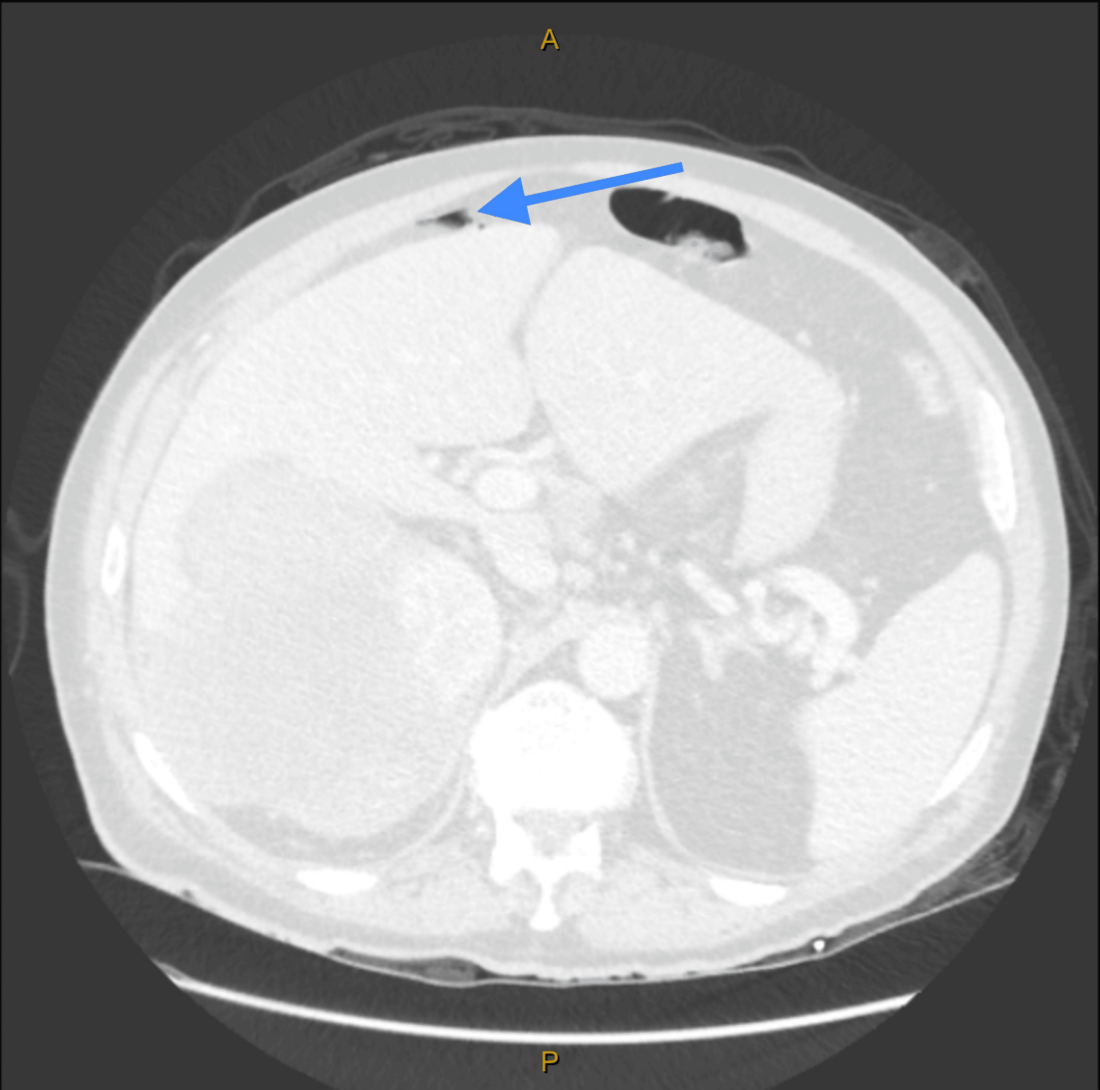
CT abdomen and pelvis with intravenous contrast demonstrating bowel perforation with pneumoperitoneum (arrow).

Unfortunately, 12 days after initiating the first cycle of immunotherapy, our patient experienced a bowel perforation in the transverse colon. A CT scan of the abdomen and pelvis demonstrated bowel perforation with pneumoperitoneum along with an interval increase in the size of the transverse colon masses, the left adrenal mass, and the right renal mass (Figure 9, Figure 10). This complication was managed conservatively by the medical oncology team, with consultation from the surgical department. After careful consideration, it was determined that a biopsy of the right kidney would not alter the patient's prognosis, and, therefore, the procedure was discontinued. Following a three-week hospital stay, the patient was discharged and was referred to the palliative care team for ongoing support.

## Discussion

Primary malignant melanoma of GIT is an exceptionally rare entity, primarily occurring in regions where melanocytes are normally found, such as the esophagus and anorectal mucosa [1,2]. However, the existence of primary melanoma in the large bowel remains controversial.

Distinguishing primary GI melanoma from metastatic melanoma is challenging. According to Sachs et al., primary intestinal melanoma can be diagnosed if it meets specific criteria, including the presence of a solitary lesion within the GIT, no evidence of metastatic disease in other organs, histological confirmation of precursor lesions or melanosis, and a disease-free interval of at least 12 months following diagnosis [10].

Despite these criteria, many experts argue that so-called primary GI melanomas may actually originate from previously regressed cutaneous melanomas. The well-documented phenomenon of melanoma regression supports this hypothesis. Additionally, some theories suggest that primary intestinal melanoma may arise from ectodermal cells or melanoblastic precursors that migrate to the gut during embryogenesis [1,11]. Alternative hypotheses propose that neural crest-derived cells reaching the intestine via the omphalomesenteric duct or heterotopic melanocytes originating from primitive stem cells may serve as potential sources of primary intestinal melanoma [1,11].

Metastatic malignant melanoma to the GIT is far more common than primary disease, with autopsy studies revealing a high incidence of secondary involvement [2,3]. The small intestine is the most frequently affected site (51-71%), followed by the stomach (27%), large intestine (22%), and esophagus (5%) [1,4]. However, clinical detection remains rare due to the nonspecific nature of symptoms, including GI bleeding, abdominal pain, obstruction, and weight loss [3,9].

Given these factors, it is generally accepted that most cases of melanoma involving the GI tract represent metastatic disease rather than a true primary neoplasm. The ability of melanoma cells to regress or remain undetectable for prolonged periods further complicates differentiation [11]. Consequently, when diagnosing GI melanoma, a thorough review of the patient's dermatologic history and an assessment for prior or regressed cutaneous lesions are essential. From a clinical point of view, both primary mucosal malignant melanoma and metastatic malignant melanoma are more aggressive than their cutaneous counterparts and have worse prognoses. The five-year survival rate for metastatic colonic melanoma is reported to be 33% [12].

In the case discussed, the history of previously excised scalp melanoma and the renal lesion implies a diagnosis of malignant melanoma metastasizing to the transverse colon. The time between the diagnosis of primary melanoma and the discovery of colonic metastasis was approximately eight years. At the time of the initial diagnosis of scalp melanoma, the disease was localized with no evidence of metastasis. Over eight years, imaging demonstrated widespread metastatic progression, involving the transverse colon, right kidney, peritoneum, and pelvic peritoneum, now classified as Stage IV (M1c). This represents a significant evolution from an early-stage, predominantly in situ, melanoma to disseminated metastatic disease. A comprehensive review by Park et al., involving 38,279 patients diagnosed with primary melanoma and subsequent large bowel metastasis over a period of 50 years, indicated that the average interval to metastasis was 5.2 years, with 42.7% of these patients developing metastasis confined to the large bowel [5]. These findings highlight the necessity of increased awareness and continual surveillance for colonic metastasis in patients with a previous history of malignant melanoma.

CT remains the prevalent initial diagnostic tool for detecting GIT metastases from melanoma, accounting for 60-70% of cases. It serves as the standard for both staging and ongoing surveillance of melanoma patients [2]. Alternative imaging modalities like PET-CT and whole-body multiparametric magnetic resonance imaging (MRI) are also in use [9]. Recent studies have highlighted PET-CT's superior accuracy for melanoma, with a sensitivity of 91% and specificity of 92% [2]. MRI is advised for specific patient groups, including pregnant individuals or those under 24 years of age [3]. Imaging can further be complemented by colonoscopy which essentially has the greatest diagnostic value as it also allows for tissue biopsy [2]. 

For both primary and metastatic colonic melanoma, surgical resection and systemic therapies, such as immunotherapy and targeted therapy, constitute the primary treatment modalities. Complete surgical removal of metastatic lesions has been associated with increased overall survival and symptomatic alleviation [1]. Ollila et al.'s study underscores this, revealing a substantial median survival advantage for patients who underwent curative resection (48.9 months) as opposed to palliative or non-surgical management (5.4 and 5.7 months, respectively) [13]. Accurate tumor staging is crucial for surgical decision-making, particularly since isolated lesions or those in proximity are more amenable to resection compared to widespread metastatic disease, where surgical removal often becomes unfeasible [8]. In carefully selected cases, most patients can expect a recovery within six weeks after surgery for metastatic GIT melanoma [1].

Immunotherapy, either as monotherapy or in combination, has been a pivotal advancement in melanoma treatment. The Checkmate-067 clinical trial, which is among the longest studies for advanced melanoma, demonstrated an improved overall survival rate in patients treated with a combination of nivolumab and ipilimumab, showing survival rates of 52%, 44%, and 26% for the combination therapy, nivolumab alone, and ipilimumab alone, respectively [7]. Research indicates that BRAF mutations are present in up to 50% of melanoma cases. Consequently, it is recommended that all patients with malignant melanoma be screened for the BRAF V600E mutation, as this opens the possibility for treatment with BRAF inhibitors, such as dabrafenib, trametinib, and vemurafenib [1].

With the advent of modern targeted immunotherapies, adjuvant therapy with immunotherapy and BRAF inhibitors has become the standard of post-surgical care for resection of malignant melanoma. Concomitantly, there has been a noted increase in immune-related adverse events, correlating with the widespread application of these therapies across various malignancies. GIT perforation is among the most severe complications associated with immunotherapy in colonic malignant melanoma patients. Despite its recognition, reported cases of such severe outcomes remain relatively few. The exact mechanisms by which immunotherapy leads to GIT perforation are not fully understood, and the standard management currently comprises surgical intervention, corticosteroids, and immunosuppressive therapy [14]. Further research is warranted to clarify causation and to refine treatment guidelines for affected patients. In the present case, the patient suffered a bowel perforation following the initiation of doublet immunotherapy and was subsequently transitioned to palliative care. It is crucial to distinguish whether the perforation resulted from ICI-induced colitis or from necrosis of the inflammatory tumoral mass, as this distinction influences treatment decisions. Given the initial colonoscopy findings, which revealed an infiltrative and partially obstructing tumor as well as an interval increase in the size of the transverse colon on a subsequent scan, the perforation was most likely caused by rapid tumor necrosis triggered by the immune response rather than classic ICI-induced colitis. While ICI-induced colitis typically presents with diffuse mucosal inflammation and ulceration, the localized perforation at the tumor site, in this case, is more indicative of necrosis secondary to treatment response. If available, histopathology could help clarify the etiology: diffuse immune infiltration beyond the tumor would support colitis, whereas necrotic tumor cells with minimal surrounding inflammation would suggest perforation due to tumor regression [15].

The occurrence of melanoma metastasizing to the GIT is rare and presents a diagnostic challenge due to its frequently asymptomatic nature. Therefore, clinicians should maintain a high index of suspicion and ensure regular surveillance of patients with a history of melanoma. Given that the patient’s initial melanoma was staged as pT1b eight years ago, the recurrence highlights the need for effective long-term surveillance strategies. While routine colonoscopy is not recommended for asymptomatic melanoma patients, periodic PET-CT scans should be considered for high-risk individuals. PET-CT has demonstrated high sensitivity and specificity in detecting melanoma metastases and remains the preferred modality for systemic surveillance. Colonoscopy should be reserved for patients with unexplained anemia, weight loss, or GI symptoms suggestive of metastatic involvement. MRI may serve as an alternative in select cases but is not routinely used for melanoma screening. This case underscores the necessity for continued vigilance in early-stage melanoma patients with features such as regression and non-brisk tumor-infiltrating lymphocytes, which may indicate a potential for late recurrence [16].

The advent of immunotherapy has broadened the scope of surgical management in these cases, necessitating a deeper understanding and integration of such treatments by surgical teams. Preventing perforation after ICI initiation in high-risk patients requires a multifaceted approach. Risk stratification is essential, as patients with pre-existing GI lesions, such as large tumors or a history of colitis, are more vulnerable [17]. Prophylactic surgical debulking could be considered for patients with a high tumor burden in the bowel and staging laparoscopy may provide additional insight into tumor infiltration before immunotherapy initiation. Adjusting the treatment approach by starting with single-agent immunotherapy, such as nivolumab alone, rather than combination therapy, may reduce the risk of severe adverse events [18]. Close clinical and radiological monitoring after treatment initiation is critical, and any early signs of obstruction, such as abdominal pain or altered bowel habits, should prompt immediate intervention. Therefore, it is essential for surgeons to acknowledge this emerging patient demographic and understand the pertinent treatment modalities.

## Conclusions

GIT metastatic melanoma is rare and often undiagnosed due to nonspecific symptoms, emphasizing the need for a high index of suspicion, especially in patients with a history of cutaneous melanoma. This case highlights the challenge of balancing treatment efficacy with the risk of severe complications, as the patient developed bowel perforation within 12 days of initiating immunotherapy, necessitating a shift to palliative care. Histopathology and imaging were crucial for diagnosis, as routine tumor markers remained unremarkable. Given the increasing use of immune checkpoint inhibitors, early recognition of GI metastases and proactive management of potential complications are essential. Screening protocols for high-risk melanoma patients may aid in earlier detection, while future research should focus on identifying predictive markers for immunotherapy-induced GI toxicity. Ultimately, timely diagnosis, careful patient selection, and individualized treatment strategies are critical in optimizing outcomes for patients with metastatic melanoma to the GIT.

## References

[1] Kohoutova D, Worku D, Aziz H, Teare J, Weir J, Larkin J (2021). Malignant melanoma of the gastrointestinal tract: symptoms, diagnosis, and current treatment options. Cells.

[2] Vongvanichvathana T, Sakyanun P, Lertsanguansinc P (2022). Metastatic malignant melanoma to colon from cutaneous melanoma: a case report. Bangk Med J.

[3] Reddy P, Walker C, Afonso B (2014). A rare case of metastatic malignant melanoma to the colon from an unknown primary. Case Rep Gastrointest Med.

[4] Laoveeravat P, Wongjarupong N, Smith L, Wachtel MS, Islam S (2019). Isolated asymptomatic metastatic melanoma to the colon: a case report. Cureus.

[5] Park JS, Ng KS, Saw RP, Thompson JF, Young CJ (2018). Metastatic melanoma to the colon, rectum, and anus: a 50-year experience. Ann Surg Oncol.

[6] Weinstein D, Leininger J, Hamby C, Safai B (2014). Diagnostic and prognostic biomarkers in melanoma. J Clin Aesthet Dermatol.

[7] Wolchok JD, Chiarion-Sileni V, Gonzalez R (2021). CheckMate 067: 6.5-year outcomes in patients (pts) with advanced melanoma. J Clin Oncol.

[8] Azharuddin M, Sharayah A, Abbas SH, Belitsis K (2018). Malignant melanoma metastasizes to colonic polyp. Cureus.

[9] Serrao EM, Costa AM, Ferreira S (2022). The different faces of metastatic melanoma in the gastrointestinal tract. Insights Imaging.

[10] Sachs DL, Lowe L, Chang AE (1999). Do primary small intestinal melanomas exist? Report of a case. J Am Acad Dermatol.

[11] Serin G, Doğanavşargil B, Calişkan C, Akalin T, Sezak M, Tunçyürek M (2010). Colonic malignant melanoma, primary or metastatic? Case report. Turk J Gastroenterol.

[12] Xie J, Dai G, Wu Y (2022). Primary colonic melanoma: a rare entity. World J Surg Oncol.

[13] Ollila DW, Essner R, Wanek LA, Morton DL (1996). Surgical resection for melanoma metastatic to the gastrointestinal tract. Arch Surg.

[14] Beck TN, Boumber Y, Deneka AY (2020). Colonic perforation after dual ipilimumab and nivolumab treatment. ACS Case Rev Surg.

[15] Kim MK, Hwang SW (2024). Endoscopic findings of immune checkpoint inhibitor-related gastrointestinal adverse events. Clin Endosc.

[16] Straker RJ 3rd, Krupp K, Sharon CE (2022). Prognostic significance of primary tumor-infiltrating lymphocytes in a contemporary melanoma cohort. Ann Surg Oncol.

[17] Giesler S, Riemer R, Lowinus T, Zeiser R (2024). Immune-mediated colitis after immune checkpoint inhibitor therapy. Trends Mol Med.

[18] Mearns ES, Bell JA, Galaznik A, Puglielli SM, Cichewicz AB, Boulanger T, Garcia-Ribas I (2018). Gastrointestinal adverse events with combination of checkpoint inhibitors in advanced melanoma: a systematic review. Melanoma Manag.

